# Philosophy of the Nervous System

**Published:** 1879-10

**Authors:** 


### PHILOSOPHY OF THE NERVOUS SYSTEM.

Dr. Brown-Sequard, of Paris, has given the influence of his name, with a reputation belonging to two Continents in recognizing the importance of the general diffusion of information among the people concerning the nature of the nervous system, its powers over life and death, and the direct bearing which it has on human happiness. In the lectures which he has delivered to crowded audiences of the cultivated and refined in the large cities, in the publication of these in the leading papers of the country, he seems to have recognized the fact that it is for the good for all classes to have some acquaintance with the nervous system, its mode of action, its capabilities, its qualities and its powers ; the importance of its healthful preservation, its moderate uses, the danger of overtaxing it, whether in the brain, thought and study, and intense application in mental investigation and research ; whether, by the absorbing attention necessary to the conduct of complicated and momentous business interests, or whether, in the excessive indulgence of the appetites and propensities of the system, all these are proper subjects for popular consideration, as also the knowledge of what is moderation, what is excess, what the effects and symptoms of excess, and when these exist the absolute necessity of promptly seeking advice from men of acknowledged abilities, and who, for a long series of years, have devoted their whole time and attention to the study, the investigation and the treatment of all the maladies of this important department to accurately determine the diseased conditions, their nature, their causes, their origin, whether arising from the want of exercise, its irregularity or its excess, and who, from their experiences and observation and practice, have learned to discover at once the nature of the derangement, and as promptly apply the remedy, thus avoiding the painfully slow feeling after the truth, so common to the inexperienced, and so annoying to the patient, who very naturally wants to know, without delay, what is the matter, the extent of the derangement, the probabilities of recuperation, the time required, and the remedy to be applied.

If the intellectual department of the brain has become exhausted by intense mental application, depriving it of the power of connected thought, it can only recover its strength by being properly fed and nourished, by the use of certain systematic methods and appliances; it is precisely so in every other department, whether of appetite, propensity, or passion: each requiring a different remedy for its recuperation, and different means for bringing them up from their over-worked and exhausted conditions. The importance of this branch of medical practice can scarcely be over-rated, for it reaches even to the perpetuation of family names, the securing of hereditary titles, and the retention of estates in the same family connections.

